# The influence of social support on subjective health among people with selected citizenships

**DOI:** 10.1093/eurpub/ckac131.504

**Published:** 2022-10-25

**Authors:** M Bug, K Kajikhina, C Hövener, C Koschollek

**Affiliations:** Robert Koch-Institute, Berlin, Germany

## Abstract

**Background:**

Social support as a psychosocial resource has a significant impact on health. However, data on the association between social support and subjective health among people with a migration history is scarce. The aim of this analysis was to examine this association among a sample of people with selected citizenships in consideration of socio-demographic and migration-related factors.

**Methods:**

Data from the project “Improving Health Monitoring in Migrant Populations (IMIRA)” was used, including people with Croatian, Polish, Romanian, Syrian, and Turkish citizenship. Descriptive and logistic regression analyses were conducted to analyse the effect of socio-demographic (gender, age, socio-economic status (SES)) and migration-related factors (length of residence, residence status, German language proficiency) on the association between social support and subjective health (very good/good).

**Results:**

A total of 60.8% of participants with a low level of social support, indicated good subjective health in comparison to 78.8% among those who reported strong support. Participants with a length of residence of over 20 years (aOR=0,29) and (very) poor German language proficiency (aOR=0,39) were less likely to report good health. A high SES had the strongest impact on good subjective health (aOR=5,42).

**Conclusions:**

Overall, the results confirm the findings for the general population in a sample of people with selected citizenships. The fact that people with a migration history more often face structural and health related barriers and that the existence of resources is helpful in overcoming these, a differentiated consideration of the relationship between social support and subjective health seems necessary in order to establish targeted prevention measures.

**Key messages:**

• Among people with selected citizenships, good social support has a positive impact on subjective health.

• Results remain consistent when considering socio-economic and migration-related factors.

